# The Reciprocal Relationship between LDL Metabolism and Type 2 Diabetes Mellitus

**DOI:** 10.3390/metabo11120807

**Published:** 2021-11-28

**Authors:** Isabella Bonilha, Eric Hajduch, Beatriz Luchiari, Wilson Nadruz, Wilfried Le Goff, Andrei C. Sposito

**Affiliations:** 1 Cardiology Division, Atherosclerosis and Vascular Biology Laboratory (AtheroLab), State University of Campinas (Unicamp), Campinas 13083-887, Brazil; bella_bonilha@hotmail.com (I.B.); beatriz.m.luchiari@gmail.com (B.L.); 2 Centre de Recherche des Cordeliers, INSERM, Sorbonne Université, F-75006 Paris, France; eric.hajduch@crc.jussieu.fr; 3 Cardiology Division, Cardiovascular Pathophysiology Laboratory, State University of Campinas (Unicamp), Campinas 13083-887, Brazil; wilnj@unicamp.br; 4 Unité de Recherche sur les Maladies Cardiovasculaires, le Métabolisme et la Nutrition, ICAN, Inserm, Sorbonne Université, F-75013 Paris, France; wilfried.le_goff@sorbonne-universite.fr

**Keywords:** type 2 diabetes mellitus, low-density lipoprotein, oxidation, glycation, modified LDL, small and dense LDL, endothelial dysfunction, deleterious effects

## Abstract

Type 2 diabetes mellitus and insulin resistance feature substantial modifications of the lipoprotein profile, including a higher proportion of smaller and denser low-density lipoprotein (LDL) particles. In addition, qualitative changes occur in the composition and structure of LDL, including changes in electrophoretic mobility, enrichment of LDL with triglycerides and ceramides, prolonged retention of modified LDL in plasma, increased uptake by macrophages, and the formation of foam cells. These modifications affect LDL functions and favor an increased risk of cardiovascular disease in diabetic individuals. In this review, we discuss the main findings regarding the structural and functional changes in LDL particles in diabetes pathophysiology and therapeutic strategies targeting LDL in patients with diabetes.

## 1. Introduction

The prevalence of type 2 diabetes mellitus (T2DM) has increased consistently worldwide for several decades, shifting the overall picture of cardiovascular disease (CVD) and its pathophysiological background. The combination of disruptions in both glucose and lipid metabolism frameworks comprise the complex pathophysiology of T2DM, favoring accelerated and severe atherosclerotic disease. Dyslipidemia is observed in 72–85% of T2DM patients and is a predictor of CVD [1]. Typically, diabetic dyslipidemia is characterized by low levels of high-density lipoproteins (HDL), hypertriglyceridemia, and an increase in the proportion of small, dense, low-density lipoproteins (LDL) [2,3]. Nevertheless, several other changes in the lipid profile have been reported, particularly regarding LDL particles, which may influence the cardiovascular risk.

An essential factor for the change in LDL phenotype in T2DM is a decline in insulin sensitivity [4]. Insulin resistance increases the triglyceride content of LDL and decreases LDL particle size [5]. Additionally, in patients with T2DM, LDL particles exhibit a wide range of oxidation levels, ranging from minimally oxidized LDL (MM-LDL) to fully oxidized LDL (Ox-LDL) [6]. The degree of oxidation of LDL correlates directly with the magnitude of insulin resistance, the accumulation of visceral fat, the presence of metabolic syndrome, and T2DM duration [7,8]. Elevated Ox-LDL concentration is also associated with an increased risk of incident diabetes due to its effects on β-cells [9] and with a higher risk of developing obesity and CVD [7]. This indicates a bidirectional association between Ox-LDL and T2DM. Lastly, glycation and the change in lipid composition of LDL have also been reported to increase atherogenic potential [10]. In this review, we discuss recent advances in the evidence of biological mechanisms that underlie LDL phenotype changes during T2DM pathology and their clinical and therapeutic implications.

## 2. Low-Density Lipoproteins

LDL are particles that vary in size, density, chemical composition, and atherogenicity [11]. Apolipoprotein (apo)-B100-containing lipoprotein biogenesis begins with intrahepatic cholesterol, either from intestinal absorption or from de novo synthesis incorporated into very-low-density lipoprotein (VLDL) particles [12]. In the bloodstream, VLDL is converted by lipoprotein lipase (LPL), cholesteryl ester transfer protein (CETP) [13], and hepatic lipase [14] into cholesterol-enriched lipoproteins, such as LDL [13]. The LDL particle has an average diameter of 22 nm and comprises a 700 phospholipid-molecule surface layer as well as a molecular core, consisting of 170 triglycerides (TG) and 1600 cholesteryl esters (CE). Additionally, this lipoprotein contains approximately 600 molecules of non-esterified cholesterol [15,16], one third of which are located in the core and two-thirds on the surface [17]. Each LDL particle contains a single apoB-100 molecule composed of an amphoteric α-helical domain that contributes to the amphiphilicity of LDL particles, and a β-sheet domain that corresponds to 40% of the structure of apoB-100 and grants stability to the LDL particles [18].

The density of lipoproteins is directly proportional to their protein content [19]. With a density of 1.019 to 1.063 g/mL, LDL has a composition of 20% protein and 50% cholesterol, including both CEs and free cholesterol (FC) [19]. Traditionally, LDL can be fractionated into discrete components by density-gradient centrifugation or affinity chromatography, according to their decreasing size and increased density [19]. The largely used classification groups LDL into five subfractions [20,21], ranging from the largest and least dense to the smallest and most dense.

Insulin resistance-induced dyslipidemia is characterized by the presence of small, dense LDL (sdLDL) particles, [4] which have a more substantial atherogenic potential compared to other LDL subfractions [22]. As insulin resistance becomes more severe, the size of LDL particles decreases [23]. Moreover, sdLDL circulation times are longer due to their relatively reduced affinity to LDL receptors (LDLR) [24], secondary to apoB-100 glycation [25] (see the section below). In addition, sdLDL displays increased affinity to the arterial wall [4], contributing to atherogenesis [26].

## 3. Influences on LDL Subfraction Heterogeneity

Two LDL phenotypes were discovered in 1990 [27]. Individuals with a higher proportion of the sdLDL subfraction, also termed the “B” phenotype (particle diameter <25.5 nm) [18], had a three-fold greater risk of having a myocardial infarction when compared with individuals with a higher prevalence of larger LDL particles, termed type “A,” regardless of total LDL concentration [28]. In 1994, a study postulated that, in LDL particles, TG content *per se* does not influence the overall apoB-100 structure, whereas LDL size plays a significant role in determining apoB-100 conformation near its receptor recognition site and, thus, its affinity for the LDLR [29]. Overall, this evidence suggests that LDL particle size is more crucial than its core composition.

**Figure 1:**
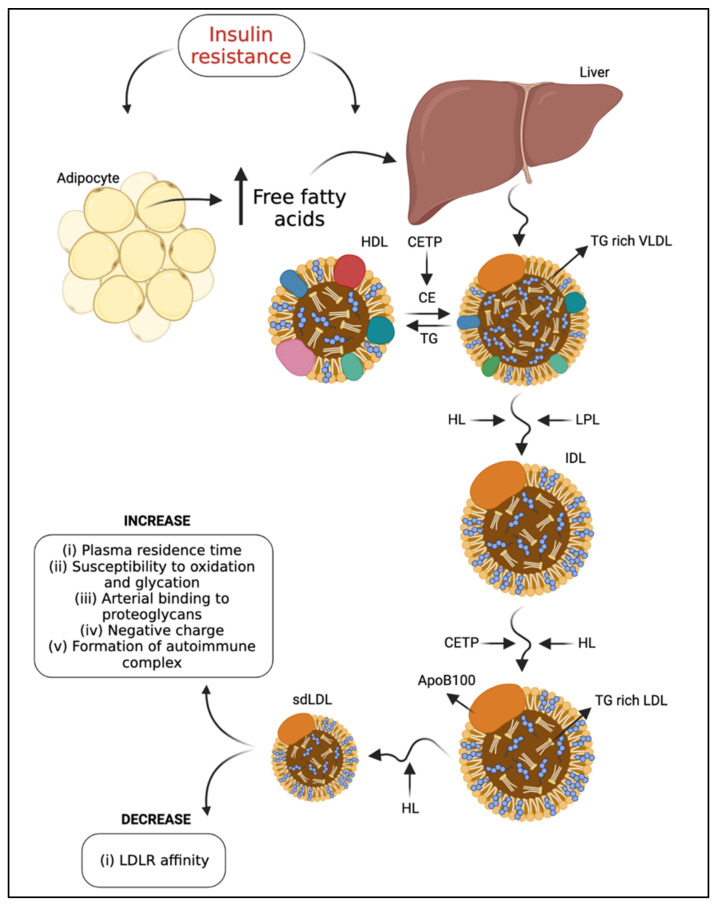
Schematic representation of the mechanism behind the generation of sdLDL in T2DM. Mechanisms leading to sdLDL in T2DM. Insulin resistance promotes triglyceride lipolysis in adipocytes and the release of free fatty acids (FFA) in circulation. Uptake and accumulation of FFA in the liver results in hepatic gluconeogenesis and affects lipid metabolism. FFAs taken up by hepatocytes are used to form new TG-rich VLDL particles. Remodeling of TG-rich VLDL through the action of CETP, HL, and LPL enzymes promotes the formation of small and dense LDL and more atherogenic particles. Consequently, these particles are electronegative, remain longer in the plasma, are more susceptible to oxidation and glycation, are more prone to bind to proteoglycans in the subendothelial space of the arterial vessel wall, and interact with beta2-glycoprotein I to form autoimmune complexes related to inflammation state. On the other hand, there is a decrease in affinity with LDLR. FFA: free fatty acid, TG: triglyceride, HL: hepatic lipase, LPL: lipoprotein lipase, CETP: cholesteryl ester transfer protein, VLDL: very low-density lipoprotein, IDL: intermediate density lipoprotein, LDL: low-density lipoprotein, sdLDL: small dense lipoprotein, LDLR: LDL receptor.

In addition, LDL metabolism is modified in T2DM. It has been suggested that there is metabolic channeling within the VLDL-intermediate density lipoprotein (IDL)-LDL delipidation cascade, so that parallel processing pathways generate different LDL products. Compared to individuals with type “A” phenotype, type “B” is associated with an approximately two-fold increase in plasma TG, higher plasma apoB-100 and IDL levels, and reduced HDL cholesterol (HDL-C) and apolipoprotein (apo) A-I concentrations [22]. These data indicate that variations in the availability of hepatic TG may determine the quantities of sequential lipoproteins [30]. Therefore, plasma levels of VLDL correlate with increased density and decreased size of LDL [5], resulting from the exchange of TG from VLDL to CE from LDL, mediated by CETP. This TG-rich LDL is a substrate for hepatic lipase [4]. In addition, CETP-mediated transfer of CE from HDL to sdLDL and VLDL-1, the precursors of sdLDL, contributes to the formation of sdLDL in T2DM and is dependent on the degree of triglyceridemia (40). The sdLDL levels were associated with a metabolic condition characterized by increased hepatic lipase concentration [31] and decreased LPL activity [32] (Figure 1). Conversely, the increase in LPL activity induced by a high-fat diet contributed to a significant increase in the large LDL I subtype concentration [32].

The sdLDL differ from other subtypes due to their binding affinity to proteoglycans, resulting from their apoB-100 conformation [33]. More specifically, sdLDL display a lower binding affinity for LDLR and, thus, a longer circulation time [34]. Plasma levels of sdLDL [35], as the proportion of small/large LDL, are significantly elevated in diabetic individuals [36]. An increase in sdLDL is a risk factor for developing atherosclerosis and coronary heart disease in patients with or without diabetes mellitus (DM), and the formation of sdLDL appears to be increased in individuals with overt insulin resistance and hypertriglyceridemia [37]. The intima-media thickness layer (IMT) of the carotid artery of diabetic patients was strongly associated with sdLDL, followed by apoB-100 and LDL cholesterol (LDL-C) [36]. These results show that sdLDL was a good lipid marker for assessing carotid atherosclerosis among the tested lipid parameters [36].

## 4. LDL Modification Due to T2DM

**Figure 2:**
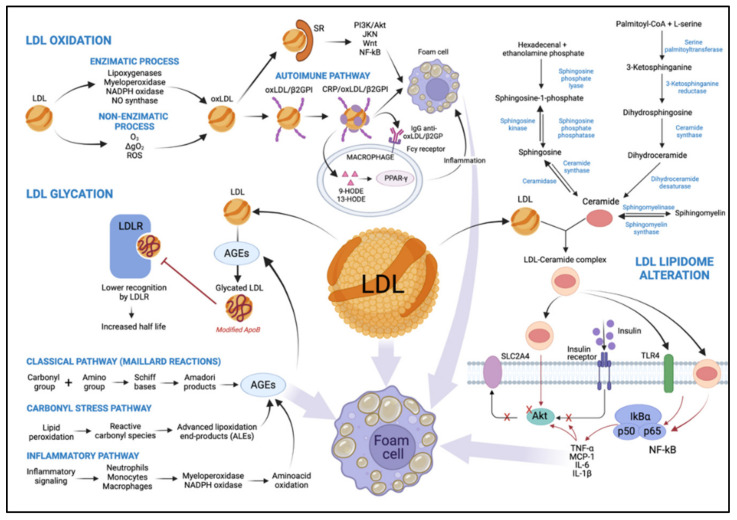
Representation of the main mechanisms that lead to LDL modification as a result of T2DM. The oxidation process can occur via two main pathways, by an enzymatic or non-enzymatic process, playing an important role in the development of atherosclerosis. Oxidation by the enzymatic process involves lipoxygenases, myeloperoxidases, NADPH oxidase, and nitric oxide synthase. Oxidation by a non-enzymatic process involves free transition metal ions. The Ox-LDL loses its affinity with LDLR and binds to SRs activating signaling pathways like Akt, JNK, Wnt, and NF-κB. It is also capable of forming a complex with β2GPI and CRP, promoting an inflammatory process. It serves as a potent regulator of macrophage gene expression involving PPAR-γ activation through 9-HODE and 13-HODE metabolites. Both mechanisms alter LDL, increasing macrophage uptake, promoting foam cell formation, and leading to more LDL modification. A higher level of AGEs is a consequence of diabetes, and it can be formed by three distinct pathways, both of which decrease affinity with LDLR, promoting an increase in half-life and contributing to the formation of foam cells. Lipidomic alteration of LDL being enriched with ceramides plays an important role in the development of tissue insulin resistance, involving insulin-signaling pathways such as IRS and Akt. They also promote the induction of transcription factors involving inflammation and the consequent formation of foam cells. O^3^: ozone, ^1^ΔgO^2^: singlet oxygen, ROS: reactive oxygen species, SR: scavenger receptors, Ox-LDL: oxidized LDL, β2GPI: beta2-glycoprotein I, CRP: C-reactive protein, LDLR: LDL receptor, 9-HODE and 13-HODE: 9- and 13-hydroxyoctadecadienoic acid, respectively, AGEs: advanced glycation end products, SLC2A4: insulin-sensitive solute carrier family 2, member 4, TLR4: toll-like receptor 4, MCP-1: monocyte chemoattractant protein-1, TNF- α: tumor necrosis factor-α, IL-6 and IL-1β: interleukin 6 and 1β, respectively.

Proatherogenic factors may also mediate other non-atherosclerotic cardiovascular changes related to diabetes, such as diabetic cardiomyopathy. Insulin resistance is the primary precursor of the pathophysiology of diabetic cardiomyopathy [38]. Studies show that insulin resistance may be related to mitochondrial dysfunction in skeletal muscle in non-diabetic and non-obese elderly patients [39] and in those with T2DM [40]. As a result of insulin resistance, intracellular lipase increases the release of non-esterified fatty acids (NEFA) from TG stored in adipose tissue. High levels of NEFA and glycogen reserves promote hepatic TG production, which is associated with increased apoB-100 secretion [41,42]. Insulin activates the phosphatidylinositol-3 kinase (PI-3K) pathway that inhibits apoB-100 secretion while activating a mitogen-activated protein kinase (MAPK) that down-regulates the microsomal TG-transfer protein (MTP) expression. This mechanism contributes to increased VLDL secretion in T2DM [16]. Therefore, in T2DM patients, TG-rich VLDLs are part of the central mechanism to generate sdLDL and reduce plasma HDL levels through CETP-mediated transfer of CE [43], as commented earlier. Interestingly, LDL binding of heparin increases in T2DM, triggering pro-atherogenic LDL modifications. Thus, the effects of heparin binding are negatively associated with atherogenesis for VLDL but are positively associated for LDL [44]. LDL enriched with TG is then a substrate for lipases linked to the endothelium, whose action leads to the formation of smaller particles depleted of lipids [34]. Additionally, LDL with high NEFA content have greater inflammatory potential and an altered structure that promotes their aggregation [45] (Figure 2).

Moreover, particles of TG-rich LDL are a preferential substrate for hepatic lipase or LPL action, resulting in lipid-depleted sdLDL [42]. In vitro, sdLDL showed reduced affinity to their receptor, a greater propensity for transport to the subendothelial space, increased arterial binding to wall proteoglycans, and susceptibility to oxidative modifications, with atherogenic potential [5]. In addition to sdLDL, a portion of LDL with increased negative charge, called electronegative LDL (−) [46], is elevated in patients with T2DM and high cardiovascular risk [47]. In anion-exchange chromatography techniques, LDL (−) exhibited high TG content, suggesting a relationship between insulin resistance and LDL (−) since glycemic control did not reduce LDL (−) in T2DM [48]. As we discuss later, all these changes indicate modifications to the recognized functions of these lipoproteins.

### 4.1. LDL Oxidation

The chemical composition of LDL makes these particles susceptible to oxidation, and atherogenic modifications exacerbate this vulnerability. The polyunsaturated acyl chains of CE, phospholipids, and TG are vulnerable to oxidative stress [49]. Most evidence suggests that oxidative modification in LDL plays a vital role in the onset of diabetes and the pathogenesis of atherosclerosis [50]. However, previous studies hint at the limitation of evaluating LDL particle oxidation indirectly through the analysis of serum autoantibodies against Ox-LDL [51,52], which does not reflect the overall extent of oxidative damage. The LDL oxidation process is described in the paragraphs below.

LDL oxidation occurs via two main pathways. Initially, oxidative changes in LDL lipids can result in the absence of, or a slight change to, apoB-100, known as MM-LDL, preserving its function as an LDLR ligand. This modified lipoprotein does not bind to scavenger receptors (SRs) [6], has a slight negative charge, activates anti-apoptotic signaling, and induces endothelial cells to express tissue factors and chemokines [53]. Subsequently, LDL lipids are oxidized and become cytotoxic and pro-apoptotic [53]. This activates inflammatory signaling pathways, leading to inflammatory cell recruitment and further LDL modification. Importantly, continuous oxidation of LDL causes modification of apoB-100. After the oxidation process is complete, Ox-LDL lose their affinity for LDLR and bind to SRs [18], such as class A1 scavenger receptor (SR-A1), CD36, lectin-like oxidized LDL receptor-1 (LOX-1), scavenger receptor expressed by endothelial cell-I (SREC), scavenger receptor for phosphatidylserine and oxidized lipoprotein (SR-PSOX), and CD68 [54]. This phenomenon results in the formation of cholesterol-loaded foam cells via PI-3K/protein kinase B (Akt)-dependent mechanisms [53] but can be upregulated via janus kinase (JNK), Wnt, and factor nuclear kappa B (NF-κB) signaling [55]. As lesions advance, a necrotic nucleus is formed in atheromatous lesions [45] (Figure 2).

Particles of Ox-LDL also interact with beta2-glycoprotein I (β2GPI), promoting the formation of the Ox-LDL/β2GPI complex as a putative autoantigen in autoimmune-mediated atherosclerotic vascular disease [56]. C-reactive protein (CRP), an acute-phase protein and predictor of cardiovascular risk [57], is associated with the complex. CRP/Ox-LDL/β2GPI complexes were found in the serum of patients with T2DM and atherosclerosis, suggesting interactions between arterial inflammation, hyperglycemia, and hypercholesterolemia [56] (Figure 2).

Several studies have revealed that Ox-LDL represent a potent regulator of macrophage gene expression [58] involved in the inflammatory response, including those encoding tumor necrosis factor (TNF)-α; the interleukins -1α, -1β, and -6; and platelet-derived growth factor (PDGF) [59]. Moreover, the oxidized lipoprotein activates peroxisome proliferator-activated receptor γ (PPAR-γ)-dependent transcription through a signaling mechanism involving scanning receptor-mediated cell uptake [60]. Two oxidized metabolites of linoleic acid, 9- and 13-hydroxyoctadecadienoic acid (9-HODE and 13-HODE, respectively), can bind and activate PPAR-γ, functioning as pathological endogenous ligands [61] (Figure 2). Consequently, these oxidized metabolites act by regulating the expression of the gene on macrophages during atherogenesis. Both free and esterified HODE metabolites are increased by 49% in the LDL of diabetic patients, reflecting a free radical peroxidation of 18: 2 n-6, in line with the decrease of 18: 2 n-6 in lipid-rich classes in polyunsaturated fatty acids (PUFA), CE, and phosphatidylcholines. Furthermore, lipid peroxidation in LDL in diabetic patients is twice as high as that in healthy patients [62]. In that regard, LDL susceptibility to oxidation is shown to be greater in hyperglycemia, as a positive correlation between Ox-LDL and glycated hemoglobin (HbA1c) concentrations in diabetics has been demonstrated [49].

More specifically, LOX-1, a type II membrane glycoprotein belonging to the C-type lectin family [63], has been identified as the primary Ox-LDL receptor. LOX-1 is mainly found in endothelial cells and plays an essential role in the pathogenesis of diabetic vasculopathy [64]. Evidence shows that LOX-1 expression is increased in the vascular endothelium of diabetic rats, which underscores its role in diabetes-associated endothelial dysfunction. Moreover, elevated glucose levels increase LOX-1 expression at the gene and protein levels [65]. In this scenario, LDL oxidation can occur by either enzymatic or non-enzymatic processes, as described in the following sections.

#### 4.1.1. LDL Oxidation by an Enzymatic Process

The enzymatic process involves many systems, including lipoxygenases, myeloperoxidase (MPO), reduced nicotinamide adenine dinucleotide phosphate (NADPH) oxidases, and nitric oxide synthase [49]. Lipoxygenases catalyze the stereospecific incorporation of one oxygen molecule into unsaturated fatty acids [66,67]. Their activity is thought to play a significant role in LDL oxidation by endothelial cells and activated monocytes [68], and macrophages [69]. The dual-specificity 12/15-lipoxygenases are implicated in oxidative modification of LDL and foam cell formation [70]. In vivo data obtained with 12/15-lipoxygenase-deficient mice crossbred to apolipoprotein E-deficient mice have established a pro-atherogenic role for this pathway [71] (Figure 2).

In vitro reaction of hypochlorous acid (HOCl), the primary oxidant generated by the MPO system, and LDL results primarily in modifications of apoB-100, with slight lipid oxidation [72]. HOCl reacts readily with the ε-amino groups of apoB-100 lysine residues, resulting in the formation of N-chloramines [72]. As a result, the LDL charge is modified, leading to the uncontrolled uptake of HOCl-modified LDL by macrophages. A small proportion of the LDL-associated chloramines break down to form aldehydes. These may be associated with the cross-linking and aggregation of HOCl-exposed LDL particles via Schiff base formation and the formation of advanced glycation end products (AGEs) [72]. ApoB-100 oxidation affects fractional LDL catabolism; the in vivo consequence of this has been shown to be a two-fold longer residence time of buoyant LDL and a greater than four-fold longer residence time of sdLDL in familial defective apoB-100 when compared to controls [73] (Figure 2).

NADPH oxidase, which produces superoxide anion (O_2_^−^) radicals, is also involved in macrophage-mediated oxidation of LDL [74]. This multi-component enzyme consists of cytosolic accessory proteins (Rac, p47phox, p67phox) that, when stimulated, associate with membrane catalytic subunits (Nox, p22phox) to facilitate O_2_^−^ generation [75]. The level of NADPH can be significantly increased when cells are exposed to mitogenic and transforming growth factors, high glucose levels, or hyperlipidemia. These signals increase reactive oxygen species (ROS) production and contribute to oxidative stress development [76] (Figure 2).

By reducing nitric oxide (NO) synthesis and availability, Ox-LDL disrupt the vessel wall balance, leading to impaired endothelium-dependent vasodilation [77], which, in turn, explains the pro-inflammatory, pro-oxidant, pro-thrombotic, and vasoconstrictor actions of Ox-LDL in the endothelium [77]. Tests performed on porcine subepicardial arterioles suggest that Ox-LDL up-regulate arginase I, which contributes to endothelial dysfunction by reducing l-arginine availability to endothelial NO synthase (eNOS) for NO production and, thus, vasodilation [78] (Figure 2).

Lipoprotein-associated phospholipase A2 (Lp-PLA2) is believed to play an atherogenic role in T2DM when linked to LDL [79]. The amount of Lp-PLA2 in sdLDL or LDL (−) is five to ten times greater than normal-sized or electropositive LDL particles [80]. Previous studies suggested that Lp-PLA2 binds with greater affinity to LDL through the conformational change that occurs in apoB-100 [80], due to glycation, for example.

#### 4.1.2. LDL Oxidation by Non-Enzymatic Process

The non-enzymatic process of LDL oxidation involves free transition metal ions in the catalyzation of lipid peroxidation. The most common are those initiated by the O_2_^−^/hydrogen peroxide/hydroxyl radical system of NO and by non-radical ROS such as singlet oxygen (^1^ΔgO_2_) and ozone (O_3_) [81]. LDL exposure to ROS may result in chemical damage by generating lipid peroxides and, ultimately, apoB-100 protein adducts. The oxidative modification of apoB-100 changes its ligand properties and marks its removal by SRs [52]. The degree of LDL modification is shown to be directly proportional to the rate of O_2_^−^ production by cells [82]. Arterial smooth muscle cells in culture generate O_2_^−^-modified LDL by an O_2_^−^-dependent free radical process catalyzed by Fe or Cu. This process results in increased uptake of modified LDL by macrophages, and, thus, foam cell formation and atherogenesis [82] (Figure 2).

While LDL are oxidized, an increase in O_2_^−^ production occurs, accompanying a time- and concentration-dependent decrease in phosphorylation of eNOS in Thr^495^ [83]. Furthermore, protein kinase C (PKC), which phosphorylates the Thr^495^ residue, showed diminished activity in cells incubated with Ox-LDL, which induced dissociation of eNOS and Golgi membranes [83].

### 4.2. Glycated LDL

AGEs synthesis begins with a condensation reaction between the carbonyl group of glucose and the primary amino groups of lipoproteins. The Schiff bases formed are subsequently transformed into Amadori products and other irreversible adducts [84]. Another complex class of compounds, the advanced lipoxidation end-products (ALEs), result from successive oxidation cascades. ALEs are formed by reactive carbonyl species derived from a lipid peroxidation process, such as malondialdehyde, 4-hydroxynonenal, and acrolein, detected in inflammatory and oxidative-based diseases [85] such as T2DM (Figure 2). The half-life of LDL in the bloodstream ranges from 2.5 to 3.5 days. However, it takes 6 to 7 days for non-enzymatic glycosylation to occur. Together, these data suggest that glycation occurs in the LDL particles retained in the arterial wall for longer than their in-circulation lifespan [45].

The primary amino acid of apoB-100 that undergoes glycation is lysine. About 2–17% of its residues are glycated [86], which continues with the formation of sugar–amino-acid adducts [84]. The lysine residue is essential for specific recognition by the LDLR. Thus, glycation of this amino acid promotes an increase in the average half-life of glycated LDL (Figure 2). In this scenario, circulating glycated LDL levels are increased [87] approximately two-fold [88] in diabetic patients with concomitant hyperglycemia compared to non-diabetics [87]. In vitro, glycated LDL have impaired binding to fibroblasts and greater glycation of lysine, resulting in lower recognition by the LDLR [89] (Figure 2). Regarding LDL subfraction phenotypes, it was observed that oxidized, glycated, and LDL (−) were increased in the T2DM group with poor glycemic control. In these patients, the prevalence of phenotype “B” was associated with increased oxidized LDL and glycated LDL activity, compared to phenotype “A” [90]. Furthermore, metformin therapy in T2DM patients decreased the oxidative damage mediated by AGEs derived from arginine and methionine sulfoxide [91]. Metformin therapy also showed lower dicarbonyl-derived AGEs residues than patients who did not receive metformin therapy [91].

### 4.3. Alteration of LDL Lipidome in T2DM: Ceramides

Saturated fatty acids and specific sphingolipids such as sphingomyelin can serve as precursors to ceramides (CER). CER, central lipids of sphingolipid metabolism, consist of a sphingoid base linked to a fatty acid via an amide bond. While these lipids play an important structural role in cell membranes, they are also involved in many processes such as the regulation of apoptosis, cell differentiation, and insulin signaling [92]. CER are mainly synthesized through three distinct pathways: the sphingomyelinase, recycling, and de novo pathways (Figure 2). The predominant synthesis pathway observed in conditions of obesity and lipid excess is the de novo synthesis pathway [92]. CER produced de novo (especially C16 and C18 CER species) have been well described as playing a crucial role in the development of tissue insulin resistance through targeting several components of the insulin signaling pathway, including insulin receptor substrates (IRS) and Akt [92,93] (Figure 2).

Studies have also reported an increase in circulating CER concentrations in obese T2DM patients compared to plasma CER concentrations in healthy individuals [94]. There is clear clinical evidence that circulating CER are mainly secreted by the liver and correlate with systemic insulin resistance [95]. A study in which more than 1500 American non-diabetic subjects from the multiethnic “Dallas Heart Study” cohort were observed for over seven years demonstrated a positive correlation between plasma concentrations of CER and insulin resistance [96]. In addition, circulating concentrations of dihydroCER, CER lipid precursors, are significantly increased in individuals who develop diabetes [97], up to nine years before the onset of the initial symptoms of the disease [98].

Circulating CER are mainly associated with lipoproteins in the circulation (90% of circulating CER), and 60% of CER are found in LDL in healthy individuals [95]. Interestingly, evidence shows that an increase in plasma LDL-CER in T2DM and the prevalence of obesity correlate with the severity of insulin resistance and elevated plasma TNF-α levels [94] but not with the degree of obesity [99] (Figure 2). The main CER species present in LDL is C24 CER [95]. Myotubes treated with LDL artificially enriched with C24 CER exhibited reduced insulin-stimulated glucose transport and an inhibition of the insulin signaling pathway [99].

Unlike LDL, no study has suggested a role for CER transported by VLDL and HDL in insulin resistance. However, and as with LDL, C24 CER is the predominant species of ceramides found in VLDL and HDL from healthy individuals [95]. A study showed that mice whose gene encoding the VLDL receptor was invalidated and who were subjected to a fatty diet exhibited better tolerance to glucose and better sensitivity to insulin than wild mice subjected to the same diet [100], suggesting the possibility that VLDL-CER may play a role in modulating the insulin sensitivity of these animals.

### 4.4. Deleterious Effects of LDL from T2DM Patients

As mentioned above, DM is a chronic condition with inflammatory factors, such as pro-inflammatory cytokines, that play an atherogenic role. Investigation of the influence on the expression of matrix metalloproteinases (MMP) or ADAM (a disintegrin and metalloproteinase) genes by LDL from individuals with T2DM showed expression of MMP-1, MMP-9, ADAM28, ADAM17, and ADAM15 genes compared to healthy LDL in a monocytic cell line (THP-1) cultured [101]. This result suggests a prolonged inflammatory state in these patients.

LDL modified by glycation and oxidation also act on platelet arachidonic acid metabolism [102]. LDL isolated from the plasma of T2DM individuals promoted phosphorylation of platelet p38-MAPK and the production of thromboxane B and were rich in malondialdehyde [102], which contributed to platelet aggregation hyperactivation.

### 4.5. Deleterious Effects of Modified LDL from T2DM Patients

As previously cited, Ox-LDL contribute to DM progression. cAMP-responsive element modulator (CREM) expression is inducible by activating the cAMP signaling pathway. The induced transcript encodes a novel repressor, called inducible cAMP early repressor (ICER) [103]. β cells in the presence of Ox-LDL increased the abundance of ICER, which compromised the expression of insulin and anti-apoptotic islet 1 brain gene [104], and hindered insulin production and secretion [105].

The effects of Ox-LDL also extend to the loss of pericytes, an initial characteristic of diabetic retinopathy. Human retinal capillary pericytes exposed to Ox-LDL had endoplasmic reticulum (ER) stress, resulting in mitochondrial dysfunction, apoptosis, and autophagy [106]. ER stress increased in the diabetic human retina and was correlated with the severity of diabetic retinopathy [106]. It caused dysfunction and was present in the retinas of diabetic patients, again correlating with disease severity [107]. The formation of Ox-LDL immune complexes resulted in greater toxicity for retinal capillary pericytes, decreasing their viability and increasing secretion of inflammatory cytokines, and reducing secretion of a critical anti-angiogenic factor, pigment epithelium-derived factor (PEDF) [107]. These findings enable the search for new therapies to reduce retinal damage in diabetic patients caused by Ox-LDL.

### 4.6. Endothelial Dysfunction in Diabetes by Modified LDL

Endothelial dysfunction is one of the first manifestations of T2DM and CVD. As mentioned in the previous paragraphs, the exposure of endothelial cells to hyperglycemia is accompanied by modification to their secretory action, demonstrated by the overdevelopment of the rough endoplasmic reticulum and Golgi complexes, enrichment of the intermediate filaments, enlargement of inter-endothelial junctions, and an increase in the number of plasmalemmal vesicles [84]. Together, these modifications increase endothelial cell permeability and favor the subendothelial accumulation of modified LDL [84]. Markers of endothelial dysfunction are often elevated years before any evidence of microangiopathy becomes evident [108]. Because of this, endothelial dysfunction has received significant attention in DM. Under physiological conditions, there is a balance between contraction and relaxation factors derived from the endothelium, but this can be altered in diabetes, contributing to the progression of vascular damage [108].

High glucose concentration also promotes more LDL glycation in diabetics, as shown above. In endothelial dysfunction, glycated LDL promote increased expression of adhesion molecules and modulate the fibrinolytic potential of vascular endothelial cells [109].

In addition, glycated LDL increase ROS production, which reduces the interaction between eNOS and caveolin-1, thus impairing NO production [110]. This finding was confirmed following a 24-h incubation of human endothelial cell culture with the addition of 100 µg/mL of glycated LDL that induced inhibition of eNOS expression and increased inducible NO synthase (iNOS) expression and, consequently, ROS production [111]. In vitro, the pro-apoptotic activity of glycated LDL gradually increased as the concentration increased, contributing to endothelial dysfunction [109]. Reactivity of the mesenteric arteries incubated with glycated LDL was significant decreased in the vasodilator response to acetylcholine and sodium nitroprusside [112]. These data indicate that glycated LDL inhibit endothelium-dependent relaxation.

The decrease in capillary density of the endothelium influences the development of atherogenic changes in lipoprotein concentrations through the aggravating activity of LPL bound to the endothelium. A reduced capillary endothelial surface area may impair access of TG-rich lipoproteins to LPL [110]. These lipoproteins indirectly affect endothelial function through the production of sdLDL [113]. An increase in O_2_^−^ release in endothelial cells exposed to LDL from individuals with T2MD was observed, suggesting that glycation or a difference in composition of LDL initiates this process [114]. Additionally, NO bioactivity was reduced with the addition of LDL from subjects with diabetes. This imbalance between NO and O_2_^−^ contributes to endothelial dysfunction [114]. In Goto Kakizaki rats, a model of insulin resistance, an increase in O_2_^−^ was observed in the vasculature of the abdominal aorta and nitrotyrosine, indicative of the formation of peroxynitrite [115]. These are mechanisms that reduce NO bioavailability and impair endothelial function.

Transfer of Ox-LDL across the endothelium of the endothelial wall occurs at sites of endothelial disruption and also through inflammation. In this process, Ox-LDL induce the expression of adhesion molecules such as monocyte chemoattractant protein-1 and macrophage colony-stimulating factor. Ox-LDL also increase the expression of matrix metalloproteinase-9, which causes vascular remodeling and fibrous cap rupture. Clearly, Ox-LDL promote endothelial dysfunction [116].

## 5. Potential Therapeutic Targets

Dyslipidemia in diabetic patients is a significant risk factor in the development of atherosclerotic CVD. Cholesterol-lowering therapy has become the most robust strategy to reduce major adverse cardiovascular events (MACEs) and cardiovascular mortality over a large spectrum of clinical conditions. There are several guidelines for the treatment of dyslipidemia in diabetic patients. In this review, we address them with the aim of providing the best decision about the cardiovascular risk involved.

### 5.1. Statins

Reducing the risk of CVD in primary and secondary prevention with 3-hydroxy-3-methylglutaryl coenzyme A reductase inhibitors (statins) is well established. Statins reduce serum atherogenic lipoprotein concentrations by blocking hepatic cholesterol synthesis and increasing the availability of LDLR in hepatocytes [117]. In 2010, estimates showed that only 58.2% of individuals with CVD and 52% of individuals with T2DM over 40 years old were on statin therapy [118]. Today, more diabetics use statins, with about 65% on low-intensity statin therapy [118]. In this setting, a meta-analysis suggested that with every 1 mmol/L reduction LDL-C in plasma, there was a reduction in the incidence of heart attack (13%), myocardial revascularization (19%), and stroke (16%) [119], by about a fifth in a wide range of high-risk participants, largely irrespective of the baseline lipid profile or other presenting characteristics, including DM [120]. Thus, standard doses of statins reduce LDL cholesterol by about 40%, suggesting that they might help to prevent major vascular events [120].

Most recommendations in clinical studies involve the use of moderate-intensity statins for diabetic patients over 40 years of age, with doses adjusted to reach LDL-C levels below previously accepted targets, based on the fact that the correlation between the risk of CVD and LDL-C in diabetic patients was more robust at this level [121]. Among individuals with T2DM without known vascular disease, the average risk of a major vascular event is about 2.9% per year. The absolute benefit in this group over an average of 4.3 years of statin therapy is significant [120].

In T2DM patients, rosuvastatin significantly increased LDL-apoB-100 catabolism, with positive effects on TG-rich lipoprotein catabolism and VLDL1-apoB-100 catabolism [122]. Therefore, statin therapy appears to be beneficial for individuals with diabetes, even those without overt coronary disease or high concentrations of cholesterol [123]. In addition to these effects, there is also a reduction in high-sensitivity CRP [124]. The clinical benefits observed with the use of statins go beyond what was expected with the reductions in the lipid profile alone, suggesting pleiotropic effects of the statins. Statins increase NO bioavailability and enhance endothelium-dependent relaxation by inhibiting the production of ROS [125] that improve endothelial function and decrease vascular inflammation and oxidative stress.

### 5.2. Ezetimibe

**Figure 3:**
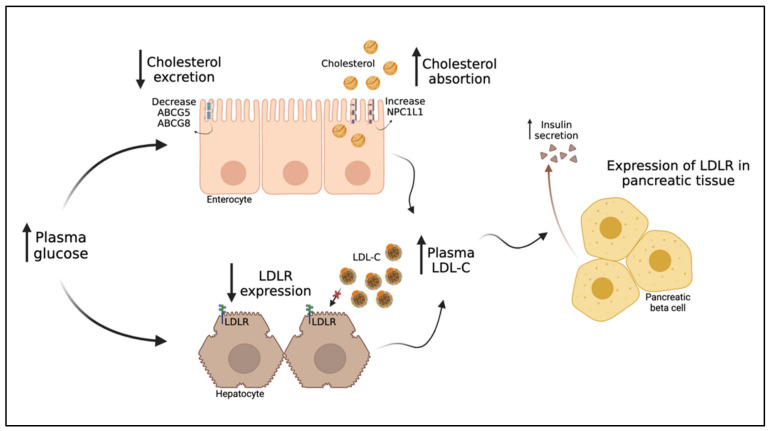
Effect of increased glucose on LDL-C metabolism and insulin secretion. In enterocytes, the increase in plasma glucose causes a decrease in cholesterol excretion consecutive to the reduction in the expression of ABCG5 and ABCG8. On the other hand, there is an increase in cholesterol absorption as a result of the high expression of NPC1L1. Both mechanisms contribute to raised plasma LDL-C levels. The increase in plasma glucose also reduces expression of LDLR in hepatocytes, another mechanistic pathway contributing to the increased plasma concentration of LDL-C. Such elevated plasma LDL-C levels together with the high expression of LDLR in pancreatic beta cells stimulate insulin secretion [2]. LDLR: LDL receptor, ABCG5/8: ATP Binding Cassette Subfamily G Member 5/ABCG8: ATP Binding Cassette Subfamily G Member 8, NPC1L1: Niemann–Pick C1-like 1 protein, LDL-C: LDL cholesterol.

Cholesterol absorption inhibitors, such as ezetimibe, appear to increase insulin sensitivity in patients with insulin resistance. They promote a moderate reduction in sdLDL and a more significant reduction in intermediate and large-sized LDL subfractions [126]. Their mechanism of action involves the inhibition of intestinal cholesterol absorption by selectively blocking the Niemann–Pick C1-like 1 (NPC1L1) protein in the brush border of jejunal enterocytes, an integral part of the uptake of micelles from the intestinal lumen to the enterocyte [127]. Ezetimibe reduces enterocyte cholesterol absorption, chylomicron formation and secretion, and cholesterol reflux from bile and increases LDLR expression on the surface of hepatocytes, resulting in reductions in serum LDL-C levels [127]. In subjects with T2DM, combined ezetimibe and statin therapy were found to reduce LDL-C and TC more than statin alone [128]. Intestinal cell cultures also demonstrated that high glucose levels heightened the expression of NPC1L1 and, consequently, cholesterol uptake [129,130]. According to these findings, diabetic patients have a higher expression of NPC1L1 mRNA than those without diabetes [131] (Figure 3).

The Improved Reduction of Outcomes: Vytorin Efficacy International Trial (IMPROVE-IT) showed that adding ezetimibe to statin therapy was beneficial in patients with T2DM; over time, there was a significant incremental reduction in median LDL-C (by 3 mg/dL) [132]. The best clinical outcome was observed in the group of individuals with a lower mean dose of simvastatin and whose additional LDL-C reduction was driven by ezetimibe [133].

### 5.3. Anti-PCSK9 (Proprotein Convertase Subtilisin/Kexin 9) Antibody (ab)

This injectable monoclonal antibody has been indicated for treating adults with heterozygous familial hypercholesterolemia or those with clinical atherosclerotic CVD who require further LDL lowering [134].

When added to a background statin treatment, anti-PCSK9ab resulted in a 42% decrease in total plasma cholesterol and a profound, near 80%, decrease in LDL-C, with a 53% reduction in total plasma apoB-100 [135]. Plasma levels of ApoC-III, a physiological inhibitor of LPL activity, have been reported to be markedly higher in T2DM patients. Glucose induces the transcription of ApoC-III, a mechanism that links hyperglycemia, hypertriglyceridemia, and CVD in patients with T2DM [136]. However, the addition of anti-PCSK9ab moderately reduced plasma ApoC-III levels (15%), and there was a more pronounced decrease in apoE concentration (33%) [135]. The prospective randomized clinical trial EXCEEDBHS3 was designed to investigate the effect of adding anti-PCSK9ab to sodium-glucose cotransporter 2 inhibitor (SGLT2i) therapy (which is reported to increase LDL levels) [137] on LDL subfractions; the trial is still ongoing [138]. In an animal model, treatment with anti-PCSK9ab reduced the size of atherosclerotic plaques and infiltration of pro-inflammatory macrophages, in addition to increasing circulating endothelial progenitor cells and angiogenic cells, suggesting that these results are secondary effects of LDL-C reduction [139].

### 5.4. Increase LDL-C with SGLT2 Inhibitors

SGLT2i is associated with a decrease in cardiovascular events and the progression of chronic kidney disease [140]. The inhibitors act by reducing renal tubular reabsorption of glucose without inducing insulin release. One study showed that empagliflozin was associated with small increases in LDL-C in T2DM patients at high risk for cardiovascular events [141]. Furthermore, a meta-analysis involving 38 studies with SGLT2i showed that canagliflozin increased LDL-C to a greater extent than other inhibitors [142]. This LDL-C increase with canagliflozin was also seen over 104 weeks in patients with inadequately controlled T2DM [143]. Conversely, in Japanese patients on a 16-week treatment regimen, LDL-C levels were indistinguishable between placebo and canagliflozin groups [144]. Dapagliflozin also slightly increased LDL-C similar to the other SGLT2i, despite having no change in the LDL:HDL ratio, which was considered unlikely to be clinically significant, according to the authors [145].

### 5.5. Insulin Treatment

Insulin therapy is widely used in patients at an advanced stage of T2DM and inefficient glycemic control, having beneficial effects on triacylglycerol and HDL-C levels [146]. Thus, to precisely analyze the effect of insulin treatment on the metabolism of lipoproteins containing apoB-100, a kinetic study of a stable isotope was performed in vivo. ApoB-containing lipoprotein metabolism was impaired in T2DM patients prior to insulin therapy [147]. Interestingly, the LDL catabolic rate was significantly decreased in patients, thereby lengthening the intravascular time. However, insulin therapy restored this catabolism to average rates, leading to a standard LDL particle residence time [147]. As already mentioned in this review, the residence time of LDL particles in plasma increases the probability of them becoming oxidized and glycated lipoproteins.

The use of intensive insulin therapy administered to poorly controlled diabetic patients using sulfonylureas caused an increase in LDL particle size and significantly reduced sdLDL levels. There was also a reduction in ApoC-III [148]. In diabetic patients with poor metabolic control, the LDLR on the surface of mononuclear cells was reduced by 41% prior to insulin treatment. After three months of therapy, LDLR expression increased by 57% in these individuals [149].

### 5.6. Thiazolidinediones and sdLDL in T2DM

Thiazolidinediones (TZDs) are insulin-sensitizing drugs and ligands for the nuclear receptor transcription factor PPARγ. PPARγ is expressed at high levels in adipose tissue, where it functions as a master regulator of adipocyte differentiation, and at much lower levels in other tissues [150]. It is also a key regulator of glucose homeostasis [151].

Sixty overweight T2DM patients without lipid-lowering therapy were randomized to metformin, pioglitazone, or gliclazide. LDL subfraction “3” mass and the LDL “3”-to-LDL ratio decreased with pioglitazone and metformin, and no change was observed with gliclazide [152]. These LDL “3” reductions were associated with reciprocal LDL “1” increases. Changes were independent of body weight, glycemic control, and TG [152]. Pioglitazone-combination treatment produced significant increases from the baseline for mean and peak LDL particle size. Pioglitazone plus metformin reduced apoB-100 levels [153].

However, drugs from the same class had varying effects on lipoprotein concentrations and sizes [154]. These differences are mainly driven by the partial activation of PPARα obtained with pioglitazone but not with rosiglitazone [155]. In patients with T2DM and dyslipidemia, pioglitazone reduced the total concentration of LDL particles, whereas treatment with rosiglitazone increased it. Both treatments increased LDL particle size, but treatment with pioglitazone had a greater effect [154]; yet, in a cross-over study performed in T2DM patients, rosiglitazone has shown to significantly increase in post-prandial status the levels of atherogenic sdLDL, which were by contrast reduced with the use of pioglitazone [156]. This may help to explain the adverse cardiovascular risk profile of rosiglitazone, and not of pioglitazone [157].

### 5.7. Glucagon-like Peptide-1 (GLP-1) Receptor Agonists, Liraglutide

Glucagon-like peptide-1 (GLP-1) is an incretin hormone, inactivated by dipeptidyl peptidase-4 (DPP-4), capable of stimulating insulin secretion after oral glucose administration [158]. In parallel, the use of GLP-1 agonists in diabetic individuals favors inhibition of glucagon production and decreases apoptosis of pancreatic β cells while promoting their proliferation [158].

In individuals with T2DM, a decrease in the fasting plasma concentration of apoB-100 and TG was observed after six months of treatment with liraglutide [159]. A kinetic study with stable isotopes showed an increase in the catabolism of TG-rich lipoproteins, including LDL [159]. In mice, this therapy increased LPL gene expression, reduced PCSK9 gene expression, and increased LDLR protein expression in the liver [159]. In clinical studies, changes in total and LDL-C were mild or absent [160]; yet, in T2DM patients, liraglutide was able to increase larger, more buoyant LDL with a concomitant significant reduction in sdLDL [161]. It has therefore been suggested that liraglutide has a strong beneficial effect on the quality rather than the quantity of LDL, with a direct anti-atherosclerotic effect [162]; this may help to explain the beneficial cardiovascular outcome of liraglutide, for which the exact mechanisms are still not fully elucidated [160].

## 6. The Intriguing Inverse Relationship between LDL-C and T2DM

### 6.1. The Inverse Relationship between LDL-C and T2DM Risk

Although the association between dyslipidemia and T2DM is well known, the causal relationship remains debatable. Evaluation of genetic variants to investigate the relationship between circulating lipid fractions and T2DM revealed a strong association between genetically lower circulating LDL-C levels and the risk of T2DM [163]. Furthermore, in an observational study of individuals not using antihypertensive or lipid-lowering therapies, low LDL-C concentrations appeared to be associated with or causing an increased risk of diabetes [164]. Regardless of lipid-lowering therapies, low LDL-C concentrations have been associated with a two-fold increased risk of T2DM, an association proportional to lifetime exposure to these low concentrations [165]. Incident diabetes did not increase when the target LDL-C level was higher than 2.59 mmol/L, meaning that the lower LDL-C target statin level contributed to the higher risk of diabetes [166].

In the Justification for the Use of Statins in Primary Prevention (JUPITER) trial, the risk of T2DM was higher in patients with LDL-C <30 mg/dL compared to those with higher levels [167]. In a meta-analysis of 13 randomized controlled trials, there was a 9% increased risk for incident DM [168]. We observed a similar result when considering the trials with anti-PCSK9ab [169]. An analysis of five studies showed that intensive-dose statin therapy was associated with an increased risk of newly acquired diabetes compared with moderate-dose statin therapy [170]. The same was found in an analysis involving eight trials with intensive statin therapy—there was an 18% higher risk of incident DM [171]. Indeed, high-dose statin therapy increased the incidence of new-onset diabetes among patients with two to four risk factors for diabetes [172]. Despite this slight increase in incident T2DM, the risk is outweighed by the reduced incidence of coronary events [168].

The molecular mechanisms mediating the association between statin use and the incidence of DM remain unclear, especially regarding the increased risk with greater reductions in LDL-C. One possible explanation involves the relationship between β-cell inflammation, oxidation, and apoptosis [173] due to the internalization of cholesterol in pancreatic β cells, resulting in impaired insulin secretion [174]. It is possible that the effect of statins on incident diabetes may originate in impaired insulin secretion, resulting in altered glucose metabolism [175], as high levels of intracellular cholesterol are detrimental to pancreatic β cells function [174]. This was verified in in vitro studies that demonstrated that prolonged exposure of isolated rat islet β cells to LDL caused necrosis [176].

In adipocytes, insulin stimulates glucose uptake by activating the insulin receptor tyrosine kinase, which in turn phosphorylates IRS-1, resulting in the recruitment of insulin-sensitive solute carrier family 2, member 4 (SLC2A4) to the plasma membrane [177]. In an animal model of T2DM, treatment with atorvastatin at clinical doses inhibited adipocyte differentiation and decreased SLC2A4 expression, impairing insulin sensitivity [177]. The reduction in SLC2A4 expression favors insulin resistance. This mechanism could explain the incidence of DM with a reduction of LDL.

### 6.2. PCSK9 and HMGCR Variants Associated with LDL-C Reduction an Increased Risk of Diabetes

The effect of anti-PCSK9 in increasing the risk of diabetes, as it markedly lowers LDL-C, was assessed using genetic scores. The study included 112,772 participants from 14 prospective cohort or case-control studies [178]. Genetic variants that mimic the effect of PCSK9 inhibitors had a very similar effect on the risk of cardiovascular events and diabetes. In addition, when statin variants were present, they showed independent and additive effects on both risks [178].

In a Mendelian randomized study, four SNPs in or near *PCSK9* were associated with a reduction in LDL-C and an increased risk of diabetes together with a higher circulating glucose concentration, body weight, and waist-to-hip ratio [179]. The presence of a defective allele in the 3-hydroxy-3-methylglutaryl-coenzyme A reductase (HMGCR) gene, which encodes the statin target, was consistently associated with an increased risk of T2DM, at least partially mediated by increased body weight [180].

The effect of DNA sequence variations that reduce plasma LDL-C levels on the incidence of coronary events was evaluated over a 15-year period [181]. The data showed that average lifetime LDL-C reduction was associated with a substantial reduction in the incidence of coronary events, even in populations with a high prevalence of non-lipid-related cardiovascular risk factors [181]. Despite the increased risk of diabetes, the benefit of decreasing the risk of CVD outweighs the other effects.

### 6.3. Reduced Risk of Diabetes in Familial Hypercholesterolemia

To verify whether the prevalence of T2DM is decreased in patients with familial hypercholesterolemia (FH), an observational study involving the Dutch FH screening registry found that the prevalence of T2DM was significantly lower in FH patients compared to unaffected relatives [174]. The prevalence of T2DM in patients with heterozygous FH was 40% lower than that observed in the general population, and LDL-C concentrations were shown not to be risk factors [182]. An inverse dose–response relationship has also been observed between the severity of familial mutation-causing hypercholesterolemia and the prevalence of T2DM [174].

## 7. Conclusions

LDL is a term comprising a set of lipoproteins that differ in size, composition, density, and atherogenicity, and whose phenotype is determined by the presence of several clinical conditions. Hyperglycemia promotes increased enteral cholesterol absorption and reduces LDLR expression in the liver. The subsequent increase in LDL-C plasma concentration stimulates glucose-mediated insulin secretion. Thus, in the short term, variations in plasma LDL-C concentration are expected when there is a disruption in glucose homeostasis. In contrast, in T2DM, chronic exposure to hyperglycemia and insulin resistance triggers a wide range of changes in LDL, in particular, the formation of sdLDL, which potentiate the pro-atherogenic action LDLs. In addition, sdLDL have reduced affinity for LDLR and, therefore, exhibit prolonged residence time in plasma, during which they are subject to constant oxidation and glycation, making them more atherogenic.

Given the above, it is evident that the progression of atherosclerotic disease occurs earlier in individuals with T2DM and that changes in LDL metabolism contribute to this predisposition. In this sense, LDL-lowering therapies, including drug combinations not previously thought to be capable of reducing levels, have been stressed in patients with T2DM with the aim of achieving intensive LDL-C goals. However, the residual risk remains high in T2DM patients, which may at least in part reflect a suboptimal change in the concentration of modified LDL particles, a condition not inferred from the usual LDL-C dosage. In this review, we presented T2DM-mediated modifications in the LDL phenotype and their potential clinical impact. The data indicate that new parameters related to LDL should be investigated as possible markers of residual risk and even as potential therapeutic targets.

## Abbreviations

T2DM	Type 2 diabetes mellitus
CVD	Cardiovascular disease
apoB-100	Apolipoprotein B-100
HDL	High-density lipoprotein
LDL	Low-density lipoprotein
MM-LDL	Minimally oxidized LDL
Ox-LDL	Oxidized LDL
VLDL	Very-low-density lipoprotein
LPL	Lipoprotein lipase
CETP	Cholesteryl ester transfer protein
TG	Triglycerides
CE	Cholesteryl esters
FC	Free cholesterol
sdLDL	Small and dense LDL
LDLR	LDL receptors
IDL	Intermediate-density lipoprotein
apoA-I	Apolipoprotein A-I
HDL-C	HDL-cholesterol
DM	Diabetes mellitus
IMT	Intima-media thickness layer
LDL-C	LDL-cholesterol
NEFA	Non-esterified fatty acids
PI-3K	Phosphatidylinositol-3 kinase
MAPK	Mitogen-activated protein kinase
MTP	Microsomal TG-transfer protein
SRs	Scavenger receptors
SR-A1	Class A1 scavenger receptor
LOX-1	Lectin-like oxidized LDL receptor-1
SREC	Scavenger receptor expressed by endothelial cell-I
SR-PSOX	Scavenger receptor for phosphatidylserine and oxidized lipoprotein
AKT	Protein kinase B
JNK	Janus kinase
NF-κB	Factor nuclear kappa B
β2ΓΠΙ	Beta2-glycoprotein I
CRP	C-reactive protein
TNF-α	Tumor necrosis factor-α
PDGF	Platelet-derived growth factor
PPAR-γ	Peroxisome proliferator-activated receptor γ
9-HODE	9-hydroxyoctadecadienoic acid
13-HODE	13-hydroxyoctadecadienoic acid
PUFA	Polyunsaturated fatty acids
HbAIC	Glycated hemoglobin
MPO	Myeloperoxidase
NADPH	Nicotinamide adenine dinucleotide phosphate
HOCl	Hypochlorous acid
AGEs	Advanced glycation end products
O_2_^−^	Superoxide anion
ROS	Reactive oxygen species
NO	Nitric oxide
eNOS	Endothelial NO synthase
Lp-PLA2	Lipoprotein-associated phospholipase A2
^1^ΔgO_2_	Singlet oxygen
O_3_	Ozone
PKC	Protein kinase C
ALEs	Advanced lipoxidation end-products
CER	Ceramides
IRS	Insulin receptor substrates
MMP	Metalloproteinases
THP-1	Monocytic cell line
CREM	cAMP-responsive element modulator
ICER	Inducible cAMP early repressor
ER	Endoplasmic reticulum
PEDF	Pigment epithelium-derived factor
iNOS	Inducible NO synthase
MACEs	Major adverse cardiovascular events
NPC1L1	Niemann–Pick C1-like 1 protein
SGLT2i	Sodium-glucose cotransporter 2 inhibitor
GLP-1	Glucagon-like peptide-
DPP-4	Dipeptidyl peptidase-4
SLC2A4	Insulin-sensitive solute carrier family 2, member 4
HMGCR	3-hydroxy-3-methylglutaryl-coenzyme A reductase
FH	Familial hypercholesterolemia
